# INFRAFRONTIER: mouse model resources for modelling human diseases

**DOI:** 10.1007/s00335-023-10010-7

**Published:** 2023-07-19

**Authors:** Asrar Ali Khan, Gema Valera Vazquez, Montse Gustems, Rafaele Matteoni, Fei Song, Philipp Gormanns, Sabine Fessele, Michael Raess, Martin Hrabĕ de Angelis, the INFRAFRONTIER Consortium

**Affiliations:** 1 INFRAFRONTIER GmbH, Neuherberg, Germany; 2 Institute of Biochemistry and Cell Biology, Italian National Research Council (CNR), Monterotondo, Rome Italy; 3 Institute of Experimental Genetics, Helmholtz Zentrum München (HMGU-IEG), Neuherberg, Germany

## Abstract

Over the last decade, INFRAFRONTIER has positioned itself as a world-class Research Infrastructure for the generation, phenotyping, archiving, and distribution of mouse models in Europe. The INFRAFRONTIER network consists of 22 partners from 15 countries, and is continuously enhancing and broadening its portfolio of resources and services that are offered to the research community on a non-profit basis. By bringing together European rodent model expertise and providing valuable disease model services to the biomedical research community, INFRAFRONTIER strives to push the accessibility of cutting-edge human disease modelling technologies across the European research landscape. This article highlights the latest INFRAFRONTIER developments and informs the research community about its extensively utilised services, resources, and technical developments, specifically the intricacies of the INFRAFRONTIER database, use of Curated Disease Models, overview of the INFRAFRONTIER Cancer and Rare Disease resources, and information about its main state-of-the-art services.

**Graphical abstract:**

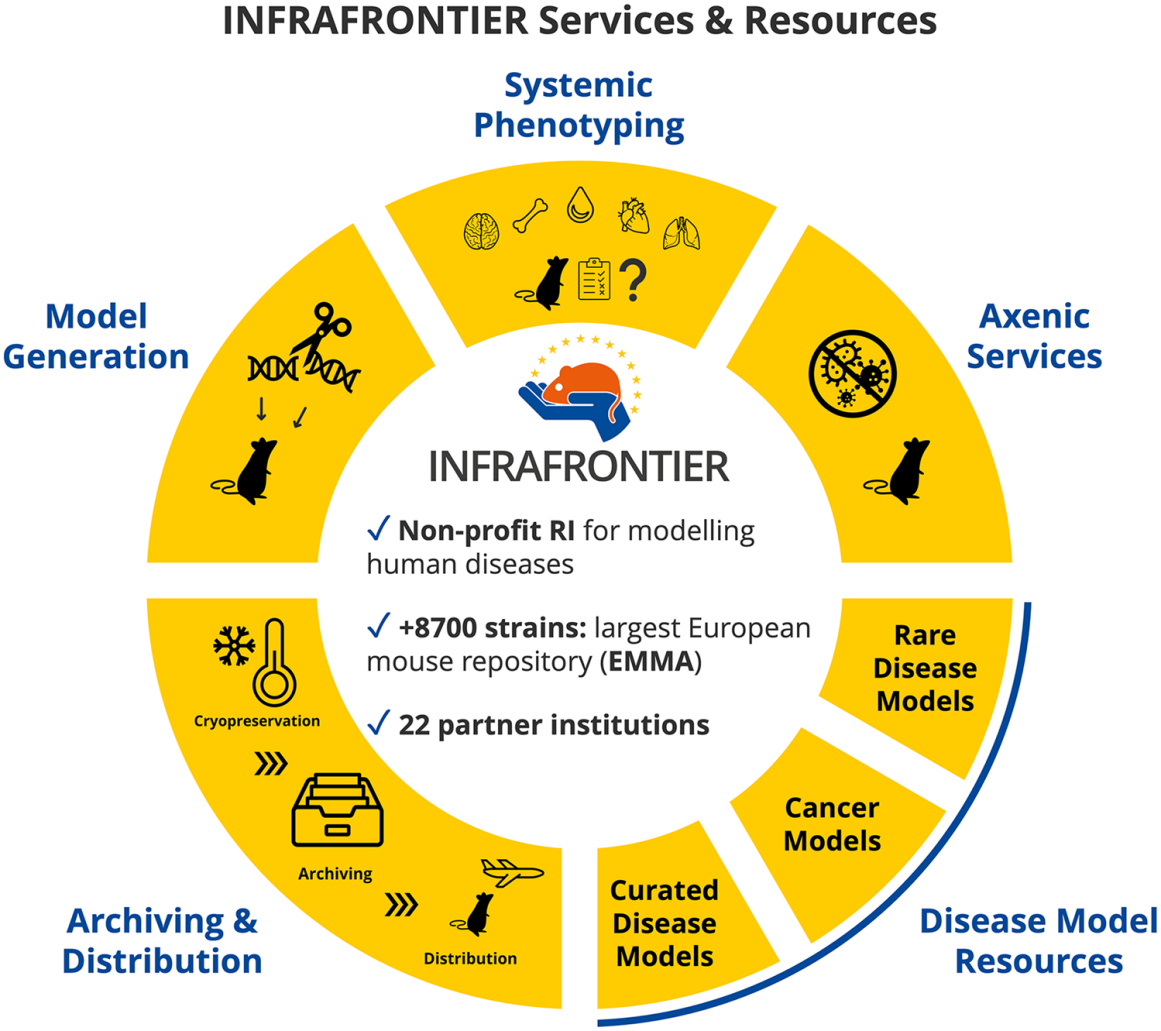

## Introduction

The importance of modelling human diseases in vertebrates, especially in mammals, lies in the need to study the causes and mechanisms involved in the development and progression of many conditions that affect humans. This only makes sense if the chosen model organism bears sufficient genetic and physiological resemblance to human beings, allowing the results to be extrapolated.

Even though there is an increasing tendency to use in vitro approaches, like organoid cultures, there are numerous studies, diseases, and syndromes that still require a complete organism to understand the underpinning molecular and physiological interdependencies. Some of these are neurological disorders, rare diseases (RDs) affecting motor function or behaviour, and, in general, diseases affecting several non-localised physiological functions (Mukherjee et al. 2022). In such cases, the local in vitro study of an affected tissue or cell type would not be able to emulate the complexity of the harmful cause or affliction suffered by the patient. The European Calcified Tissue Society recently stated that despite the advancements in in vitro and in silico models, in vivo studies are still needed to decipher the complex crosstalk between bones and tissues and the local regulation of bone physiology (Stein et al. 2023). Similarly for intestinal research, organ-on-a-chip technology is capable of imitating complex physiological features like peristalsis-like motions but still lack important features like the presence of supporting cells and tissues, gut microbiota and more (Thomas et al. 2023).

Among animal models, mice are the most widely used organisms in basic and preclinical research because of their genetic, physiological, and behavioural homologies to humans. Concerning genetics, it is estimated that 80% of mouse proteins have strict 1:1 orthologues in the human genome and about 90% of the human and mouse genomes reside within conserved syntenic segments (Mouse Genome Sequencing Consortium 2002). Moreover, many diseases have a similar symptomatology in both species, so that the study in mice yields interesting conclusions that most of the time are transferable and applicable to humans (Perlman 2016).

INFRAFRONTIER, the European Research Infrastructure for modelling human diseases, provides the scientific community with access to valuable mouse and rat strains including resources and services for their generation, phenotyping, and application in specific research pipelines (www.infrafrontier.eu). INFRAFRONTIER archives and distributes transgenic lines originally produced by individual scientists or phenotyping-dedicated platforms, such as the International Mouse Phenotyping Consortium (Groza et al. 2023) (IMPC; www.mousephenotype.org) via the European Mouse Mutant Archive (EMMA), which currently has more than 8700 strains available for distribution to researchers. As a non-profit Research Infrastructure, the archiving of lines of interest is done for free while the distribution fees cover the archiving and distribution costs for the EMMA nodes. EMMA is a core resource of INFRAFRONTIER and is part of its expanding resources and services portfolio.

The INFRAFRONTIER database was designed to be a public scientific data platform for the worldwide biomedical research community (INFRAFRONTIER Consortium 2015). Its aim is to provide up-to-date, curated information about the genetic, phenotypic, and disease model properties, as well as bibliographic references, archiving status etc., of all the mutant mouse and rat strains that EMMA stores and distributes. The INFRAFRONTIER database is a major contributor to the International Mouse Strain Resource (IMSR; www.findmice.org/) and it has been selected as a FAIR (Findable, Accessible, Interoperable, and Reusable) resource by the international FAIRSharing community (the FAIRsharing Community et al. 2019).

Apart from EMMA, INFRAFRONTIER also provides other cutting-edge resources and services to the scientific community. These include generation of precision mouse or rat models, germ-free derivation of mouse models for gnotobiotic studies, systemic phenotyping for broader phenotypic analysis, late onset phenotyping for ageing-related disorders, and more. To improve reproducibility and replicability of animal studies, INFRAFRONTIER has also developed and published quality principles for systemic phenotyping that include 11 key principles to establish a basis for reproducible and reliable research results when it comes to phenotyping of mouse models (Ehlich et al. 2022). Recently, INFRAFRONTIER has developed and launched new services and designed dedicated landing pages bringing together resources for researchers from specific disease areas. This article outlines these latest developments and updates the status quo of INFRAFRONTIER regarding the new services, resources, and technical developments along with their relevance and impact on the scientific research community.

### The INFRAFRONTIER database: finding and accessing EMMA mouse models

As mentioned earlier, the INFRAFRONTIER database is a public data repository for all relevant information concerning the strains stored in EMMA that includes unique EMMA IDs, genetic characteristics, phenotypic descriptions, phenotype ontology annotations, disease associations, characterisation protocols, breeding history, animal welfare requirements, related publications, and strain ownerships.

Strain information is received via two main channels—scientific community and large-scale projects or programmes. The scientific community contributes on an individual basis when they submit their strains for archiving in EMMA using the EMMA on-line submission form. This information undergoes a quality check before its integration into the INFRAFRONTIER database. Automated data imports from large-scale mouse mutant programmes like the IMPC are also an important data source for the INFRAFRONTIER database. This is achieved through a fully automated import procedure with daily data synchronisation and clearing several data quality compliance steps.

In addition to storing mouse strain data provided by the producers, the INFRAFRONTIER database also enriches the stored data using different sources/procedures. Each submitted strain is automatically assigned a unique and persistent EMMA alphanumeric identifier (ID), with format “EM:xxxxx”. Expert curators identify and assign the correct names, symbols, and unique IDs, for each involved gene, allele/transgene, and genetic background of any mouse mutant strain distributed by EMMA, as defined and reported by the reference Mouse Genome Informatics (MGI; www.informatics.jax.org/) resources. This allows them to assign to each strain an international strain name, according to the rules and guidelines established by the International Committee on Standardised Genetic Nomenclature for Mice (www.informatics.jax.org/mgihome/nomen).

The data enrichment is also supported by specific automated procedures. They include the automated import of most recent mouse gene, allele/transgene and genetic backgrounds described by symbols and unique IDs from MGI’s MouseMine resource (www.mousemine.org). These procedures are essential for correct strain data curation and nomenclature assignment (see above). MouseMine is also utilised for importing Disease Ontology (DO) terms of modelled diseases and Mammalian Phenotype ontology (MP) terms (gene/allele-associated), as annotated by MGI. RD Orphanet IDs and descriptions are obtained from Orphanet.

The detailed, exploitable, and quality-controlled information present in the INFRAFRONTIER database is available on the INFRAFRONTIER website (www.infrafrontier.eu), where users can access integrated mouse strain data, search the database, and explore archiving and distribution services along with dedicated disease model resources (described below). In addition, information about EMMA strains can be accessed through IMSR, IMPC, and MGI. This tight connection with other key resources strongly promotes and expands the global dissemination and use of INFRAFRONTIER disease model resources.

### Selecting the right model for the right disease

One of the ways INFRAFRONTIER promotes the use of mouse models for human disease research is by highlighting validated, reliable, and annotated models called the Curated Disease Models. This resource allows users to browse through a list of disease models and find mutant strains available from EMMA that can be used to model a certain disease. These models are curated by experts that validate the actual correlation of allele/transgene and background combinations in any EMMA mouse mutant strain with relevant mouse models of disease, as described in peer-reviewed publications and annotated by MGI. The model datasets are frequently reviewed and updated, in accordance with latest published information.

The annotation as curated disease models is complemented by the integration and display of structured phenotyping information for each strain. This has been made possible by the implementation of the programmatic acquisition, update and public presentation of current gene- and allele/transgene-specific MP annotations pertinent to the EMMA strains, from both MGI’s and IMPC’s phenotypic data resources. These currently include more than 33,000 MGI-assigned and more than 8700 IMPC-assigned, allele-specific annotations.

The Curated Disease Models presently include over 480 distinct mouse mutant models of DO-defined human diseases, as annotated by MGI through their genotypic and phenotypic descriptions in peer-reviewed publications (www.informatics.jax.org/userhelp/disease_connection_help.shtml). These models are divided into the DO categories like cardiovascular system, endocrine system, gastrointestinal system, immune system, hematopoietic system, nervous system, and more. With this curated resource, INFRAFRONTIER makes it easier for researchers to pinpoint the right model for their research—saving time, money, and effort.

### Empowering cancer research using EMMA mouse models

Cancer is a leading cause of death in the world. In 2020, it was diagnosed in more than 19 million people worldwide and killed nearly 10 million (Sung et al. 2021). The same year, the incidence in the European Union accounted for 2.7 million new cases (https://ec.europa.eu/commission/presscorner/detail/en/IP_22_7548), and an increase of 25% is estimated by 2035 (https://ec.europa.eu/commission/presscorner/detail/en/ip_21_342).

Its high prevalence and mortality, as well as the aggressiveness that impairs the patient’s quality of life, have placed cancer at the forefront of scientific research. Nevertheless, the study of cancer in humans and in vitro is greatly restricted and, thereby, animal models emerged as a relevant alternative to surpass these limitations (Winslow and Jacks 2015). Over the past 60 years, mouse models have been indispensable in the study of carcinogenesis, the process by which abnormal cell division turns into the formation of tumours. The major advantage of mouse models lies in their ease to being genetically engineered, which has allowed researchers to generate lines that reliably recapitulate almost every type of human cancer. The access to conditional lines that allow activation of oncogenesis at the desired time and target tissue has helped researchers to understand basic concepts of cancer, as well as develop and test promising treatments (Hirst and Balmain 2004).

Leveraging its expertise in disease models, INFRAFRONTIER supports cancer research in several ways. The INFRAFRONTIER Cancer Resource currently presents EMMA strains associated with more than 50 different cancer types, selectable from a drop-down menu, with additional information like phenotypic description from MGI and EMMA strain submitters. It is designed to help cancer researchers find an EMMA strain suitable for their specific research projects. The resource also depicts whether the mouse strains are genetically associated with human cancer. It should be noted that unless explicitly stated these EMMA strains are associated to a cancer via their modified gene i.e. their genotype is not necessarily a cancer genotype. These associations were accomplished by extracting collections of cancers and associated genes from MGI and linking them to EMMA strains.

Complementing the INFRAFRONTIER Cancer Resource is the list of manually curated cancer models, which are verified mouse models for 14 different types of cancers. Based on the original reports in the associated peer-reviewed publications, these strains have been accurately and systematically curated and annotated to carry mutant alleles or transgenes. They also express phenotypes that precisely model particular cancer types.

In addition, INFRAFRONTIER also provides cancer-centric services as free-of-charge Open Calls under the European Commission Horizon Europe project: Providing Cutting-Edge Cancer Research Services Across Europe (canSERV project; www.canserv.eu). Lastly, another valuable resource for the cancer research community is the Netherlands Cancer Institute’s (NKI) embryonic stem cells (ESCs) archive from Genetically Engineered Mouse Models (GEMM). This unique GEMM-ESC archive consists of ESCs which often contain multiple modified alleles and form the basis for further genetic engineering either by Flp-recombinase-mediated integration, gene targeting or CRISPR/Cas9, allowing for the evaluation of altered target gene expression in a spontaneous tumour model (Huijbers et al. 2015).

### Expanding rare disease resources with more than 2000 mouse strains

In Europe, orphan or RDs are conditions whose prevalence is lower than 1 in 2000 people. There are more than 7000 known types that affect 350–400 million people worldwide (European Commission/Public Health) (Rare disease facts by Global Genes). An aggravating factor of these diseases is the lack of research around them, which limits their understanding and hampers the development of an appropriate treatment. Consequently, millions of patients must wait an average of 4–5 years to get a diagnosis and often remain untreated (Yan et al. 2020). This situation has made RDs a global health priority.

Due to the small patient populations suffering from RDs, animal models have become particularly helpful when it comes to investigate, develop, and test novel drugs. They are crucial to understand RDs, as they help identify their genetic bases and molecular mechanisms, as well as the physiopathology and genotype–phenotype correlations. With more than 80% of RDs caused by genetic mutations (European Commission/Public Health), the mouse model is the most commonly used organism in RD research due to its well-established technologies for genome editing and high genetic and physiological similarities with humans (Murillo-Cuesta et al. 2020). By introducing mutations in the mouse genome corresponding to human disease-associated variants, researchers can study rare phenotypes and evaluate potential targeted therapies.

The INFRAFRONTIER Rare Disease Resource presently lists up to 1670 Orphanet-annotated RDs where over 2000 mutant mouse strains with mutations in a mouse gene orthologous to a human gene involved in these diseases are available for distribution from EMMA. This enables researchers working on a specific RD to search the resource for their disease of interest and find related EMMA strains and accompanying information.

When browsing the resource, users can look for a certain RD and will see the disease description from Orphanet, a list of EMMA strains and genes of interest, peer-reviewed publications using these strains (if available) and ORPHAcode. Clicking on any of the strains will take users to the strain details page where they can see additional information about the strain and order it. In essence, all the necessary information is provided in one spot for them to make an informed decision about the applicability of an EMMA mouse strain in their research.

In addition, a similar resource allows users to explore an extensive list of publications using EMMA strains listed in the INFRAFRONTIER Rare DIsease Resource. By displaying information about publications like journal, PubMed ID (PMID), EMMA strains, Medical Subject Headings (MeSH) terms, and RDs, this resource showcases the impact of EMMA strains on (RD) research and provides peer-reviewed use cases on the applicability of specific strains.

### Generating mouse models for modelling human diseases

INFRAFRONTIER partners are world-leading biomedical research institutes that provide state-of-the-art services to external researchers under the ‘INFRAFRONTIER Services’ umbrella. One highly requested service is the ‘generation of precision mouse models’, which is mostly achieved using CRISPR/Cas9 mutagenesis technologies and entails the creation of bespoke mouse models based on the needs of the researchers. The service covers the production of a single F1 genome-edited mouse line and involves project design including the prediction of off-target sites, preparation of sgRNA’s and Cas9 mRNA/protein, and injection into zygotes to generate F0 founder mutant animals (preferably on a C57BL/6N or C57BL/6 J genetic background). Selected F0 animals are bred to produce F1 genome-edited animals. Possible allele types that can be generated are indels, exon deletions (< 10 kb), and point mutation insertions. Generally, newly developed mouse models will be made available in 2–3 months following provision of all required information to start the service. This service allows researchers that lack the necessary expertise and technology to create mouse models that precisely mimic the genetic perturbations in human diseases using the wealth of experience present in the INFRAFRONTIER consortium. To date, this service has been used to generate precision mouse models for, among other diseases, leukemogenesis, acute myeloid leukaemia, colon cancer, Shwachman–Bodian–Diamond syndrome, systemic capillary leak syndrome, and Charcot–Marie–Tooth neuropathy.

In addition to generating novel models, INFRAFRONTIER also provides new mouse models to users based on already-existing mutant models. An example for this are the models generated by the IMPC in their global effort to identify the function of every protein-coding gene in the mouse genome using different targeting strategies that have different and complementary properties to produce knockout alleles. IMPC mouse mutant strains are present and used in different allelic forms, denoted as tm1a, tm1b, tm1c, tm1d, and tm1e, and differ in their complexities and research applications (Ryder et al. 2014). Most of the strains are generated and archived as ‘knockout-first’ alleles, also called tm1a, which might not be directly useful to researchers. However, animal facilities often lack the expertise to generate other allele forms. To make these strains more useful and accessible, INFRAFRONTIER partners offer a service to convert these alleles into forms that can be directly used in specific applications, like the tm1c conditional knockouts.

### Deriving germ-free mouse models for gnotobiology

In humans and mice, the microflora roughly represents 90% of the cells that compose an individual. The phenotype associated with a specific mouse genome modification may result from disruption of the interactions between the host and its microflora. Commonly, mice are raised in the now standard Specific Pathogen-Free (SPF) conditions, which do not significantly modify the extreme diversity and number of colonising micro-organisms (Lane-Petter 1962). Gnotobiology techniques allow for the generation and maintenance of animals in a germ-free (axenic) environment and the possibility to restore specific components of the microflora. The comparative analysis of a given mouse line raised in SPF and in germ-free conditions reveals the contribution of the microflora to the phenotype associated with a specific mouse genotype. For example, this approach has been essential to discriminate between autoimmunity and inflammatory immunopathologies in various mouse models (Belkaid and Hand 2014). Germ-free animals can also help researchers to understand the host–commensal interactions during tissue regeneration, like in the intestinal epithelium (Rath and Haller 2022). Metabolic disorders are now also clearly associated with the microbiota composition (Dabke et al. 2019). The notion that the microbiota could also influence behaviour is being investigated as well (Johnson and Foster 2018). Finally, the importance of microbes in cancer and cancer therapy has been increasingly claimed in the past few years (Sepich-Poore et al. 2021). The use of germ-free/gnotobiotic technology constitutes an attractive tool to test new experimental scenarios in this expanding research area.

Over the last decade, with the help of European Commission funding (Framework Programme 7 and Horizon 2020), INFRAFRONTIER has expanded its service portfolio to support microbiome research. The INFRAFRONTIER Axenic Service to derive germ-free (axenic) mice, currently offered on a non-profit fee-for-service or collaborative basis, is primarily provided by the Axenic/Gnoto Facility of the Instituto Gulbenkian de Ciência (IGC) and will be soon provided by Mary Lyon Center at Medical Research Council (MRC) Harwell and Typage et Archivage Animaux Modèles (TAAM). These institutes are the few dedicated facilities in Europe with the required equipment and expertise for generating germ-free and gnotobiotic mice for external users. The participating axenic and gnotobiology platform supports research into host–microbiota interactions to study the role of the microbiome in health and disease. In essence, this service can be used to investigate the involvement of the microbiome in metabolism, nutrition, physiology, and immune function and highlight the mammalian microbiota’s role in the pathogenesis of diseases like cancer.

The INFRAFRONTIER Axenic Service involves germ-free (axenic) derivation of mouse strains provided by users as live animals or quality-controlled embryos or sperm. The process of rederivation to axenic conditions is presently conducted through hysterectomy/caesarean section. This method entails performing an aseptic hysterectomy (C-section) on the donor mother, followed by transferring the pups to a germ-free foster mother. Additionally, the surgical implantation of embryos into germ-free pseudo-pregnant recipient females is another technique to generate new axenic strains, which is currently under implementation. Experimental procedures like body weight and body temperature measurement, buccal swab, intraperitoneal and intravenous injections, blood collection, faeces collection, surgeries (vasectomy, and renal ischaemia–reperfusion), oral glucose tolerance test, insulin tolerance test, solid tumour size measurement, and follow-up can also be requested to be performed in the generated axenic mice. The service also facilitates the provision of suitable logistic support for the transportation of the axenic animals to the requester, if needed.

Schwartz et al.(2019) utilised this service to compare the development of skin inflammation in germ-free mice with a frame shift mutation in the filaggrin gene. So far, researchers investigating skeletal muscle degenerative diseases, bladder cancer, Parkinson’s disease, and inflammatory bowel disease have also used this service. With this service provision, INFRAFRONTIER aims to fill the gap in Europe for the availability of affordable, reliable, and non-commercial germ-free mice to academic researchers.

### Systemic phenotyping for ageing-related disorders

**Fig. 1:**
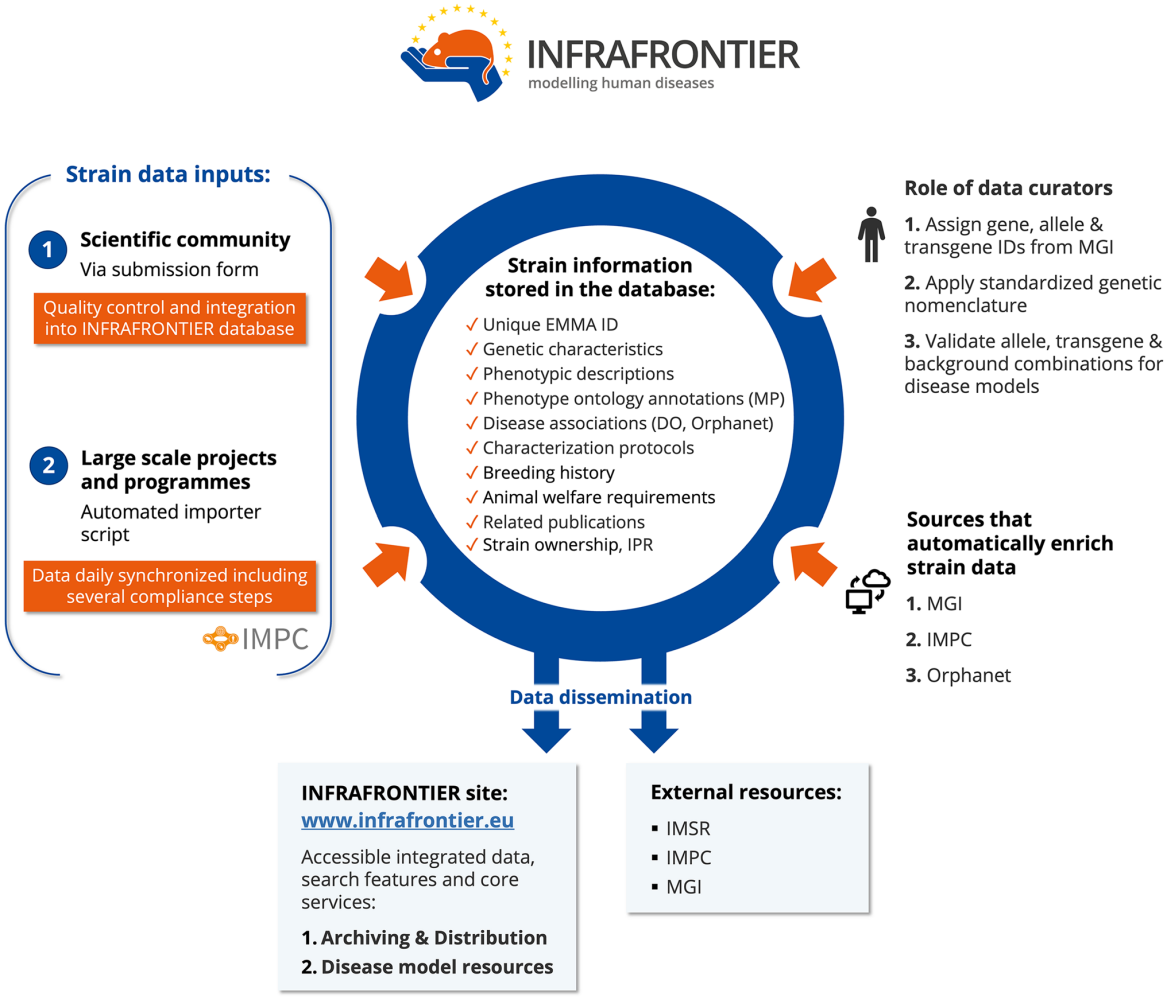
A simplified schematic representation of the data processes in the INFRAFRONTIER database that includes sources of model data, the different kinds of stored information, curation and enrichment activities, and channels for data dissemination. DO: Disease Ontology; EMMA: European Mouse Mutant Archive; IMPC: International Mouse Phenotyping Consortium; IMSR: International Mouse Strain Resource; IPR: Intellectual Property Rights; MGI: Mouse Genome Informatics; MP: Mammalian Phenotype ontology term

In ageing research, mice have also emerged as robust and dependable experimental systems. Their short life expectancy offers a unique opportunity to test genetic and therapeutic intervention strategies on ageing-related disorders and investigate their impact on lifespan and ageing indicators, within a relatively brief experimental duration. Studies on mice with an extended lifespan or those exhibiting symptoms of premature ageing, along with genetic mapping techniques, have also provided valuable insights into the underlying mechanisms driving ageing (Shcherbakov et al. 2022). Moreover, research on caloric restriction and pharmacological anti-ageing treatments in mice holds immense significance for human health (Madeo et al. 2019) (Fig. 1).

Very few laboratories in Europe are equipped to carry out a comprehensive phenotyping research project for aged mice primarily due to lack of expertise, instruments, and standardised phenotyping pipelines. First described by Gailus-Durner et al. (2009), systemic phenotyping is a broad screening pipeline where mice are analysed systematically covering a wide range of tests to identify novel phenotypes. In other words, it is the systematic analysis of mutant mice through a standardised phenotyping pipeline across a range of biological systems. This type of phenotyping is carried out in mouse clinics within the INFRAFRONTIER consortium and requires large-scale capacity, broad expertise, specific equipment, and dedicated infrastructure. This immensely useful service has been adapted within INFRAFRONTIER to aid ageing research in the form of ‘late onset phenotyping’ which is performed in early and later ages allowing exploration of late onset ageing-related diseases and was first mentioned in the INFRAFRONTIER Consortium et al. (2016). Some of the INFRAFRONTIER mouse clinics, namely the Mary-Lyon-Centre at MRC Harwell and the German Mouse Clinic (GMC), offer this specialised service allowing researchers to exploit their phenotyping expertise, technical infrastructure, and standardised pipelines. Phenotyping is performed according to the standardised experimental procedures within an agreed pipeline of tests. The tests and pipeline for the late onset phenotyping are shown below.

**Fig. 2:**
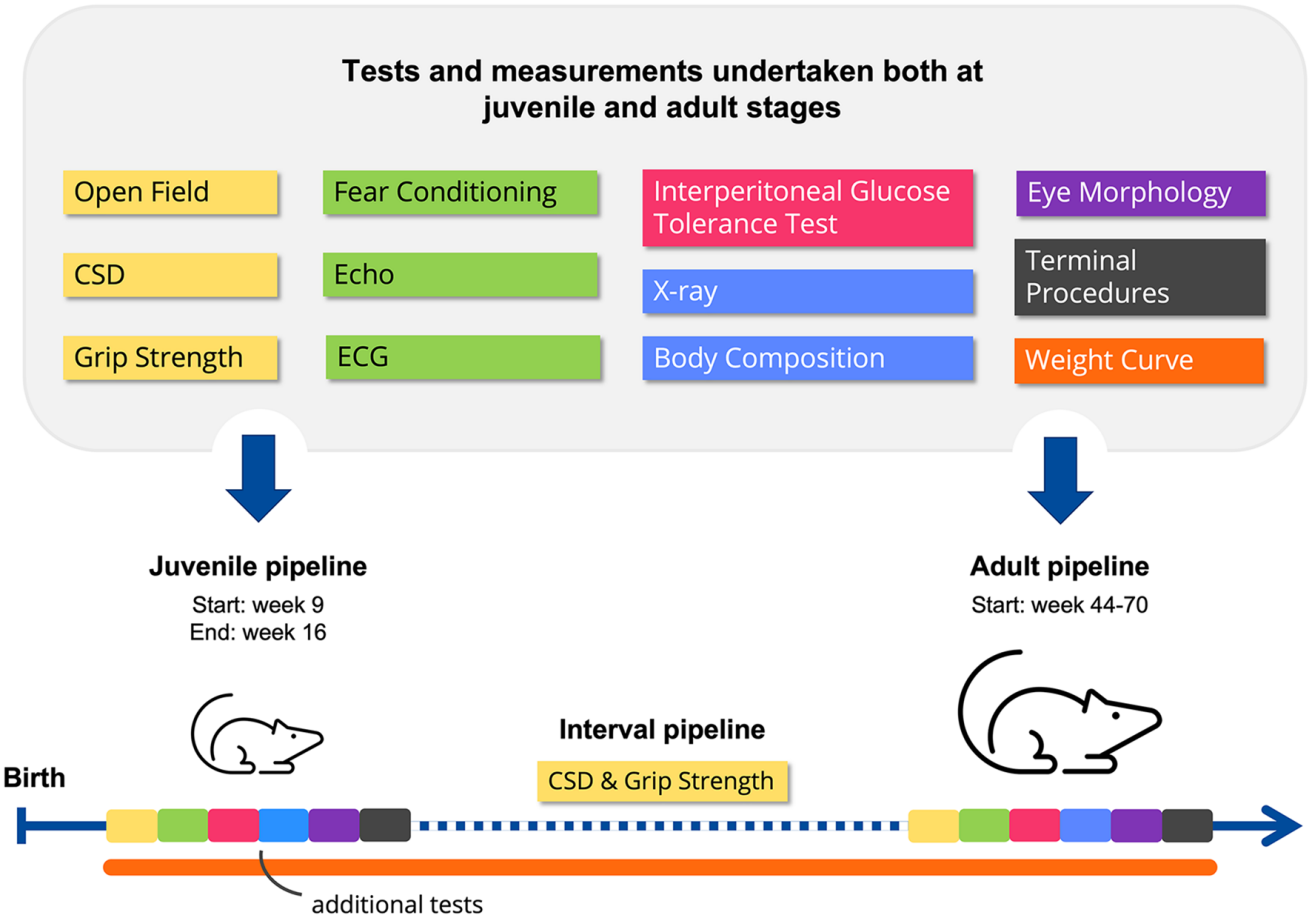
Simple overview of the late onset phenotyping carried out in INFRAFRONTIER Mouse Clinics. This phenotyping pipeline, based on the IMPC pipeline, involves standardised procedures and tests to compare juvenile and adult cohorts of mouse strains to investigate ageing-related disorders. CSD: Combined Modified SHIRPA and Dysmorphology; ECG: Electrocardiogram; Echo: Echocardiogram

Briefly, the general late onset phenotyping platform consists of distinct phenotyping sections shown in Fig. 2. The main sections of comparison are the ‘Juvenile Pipeline’ (week 9—16) and the ‘Adult Pipeline’ (after week 44), where several parameters like grip strength, echocardiogram and electrocardiogram readouts, body composition, eye morphology, etc., are measured and compared among an ageing cohort of mice strains. Additional tests and analysis like clinical blood biochemistry, tissue histopathology, immuno-phenotyping can be carried out in between these sections or at the end as terminal analyses.

Continuing the successful use cases mentioned in the INFRAFRONTIER Consortium et al. (2016), Mulderrig et al. (2021) recently used the late onset phenotyping service from MRC Harwell to show how endogenous formaldehyde accumulation drives Cockayne syndrome. Other projects utilising this service dealt with autism and behavioural anomalies, mitochondrial gene variant interactions, role of early epigenetic regulation, and assessing a novel model of Down syndrome. This specialised phenotyping service assessed the impact of complex genetic alterations on ageing and age-related phenotypes, and produced clinically and biologically important data for joint publications.

### INFRAFRONTIER today: the research infrastructure for modelling human diseases

Since its inception in 2006, INFRAFRONTIER has established itself as a valuable component of the European Research Area. Based on support from national and European research agendas, it has built a solid organisational foundation and forged rich relationships with key European and international research initiatives, such as the European Joint Programme on Rare Diseases (EJP-RD, see below) and the IMPC. INFRAFRONTIER has so far archived over 8700 mouse strains, shipped around 7000 strains, served over 15,000 user requests, and supported more than 400 research projects via European Commission funds. It has strengthened its capacities by reengineering the INFRAFRONTIER database, obtaining a licence for distributing CRISPR/Cas9-generated lines, launching successful pilot services like the COVID-19 Therapeutics Pipeline and more. Irrefutably, INFRAFRONTIER has significantly contributed to global biomedical research by providing reliable mouse strains, dependable data resources, and cutting-edge services.

Currently, INFRAFRONTIER is involved in several initiatives to bundle together resources and services from different European Research Infrastructures in the life sciences for different disease fields. Some of the notable large-scale projects include the EJP-RD (https://www.ejprarediseases.org/), ISIDORe (Integrated Services for Infectious Disease Outbreak Research, https://isidore-project.eu/), and canSERV (https://www.canserv.eu/). INFRAFRONTIER Rare Disease Resource will be findable and accessible via the EJP-RD Virtual Platform, a platform being developed under the EJP-RD project to discover, query, and access open RD resources. In ISIDORe, INFRAFRONTIER offers services for the generation of suitable mouse strains and pipelines to test therapeutics and vaccine candidates (like for COVID-19), while in canSERV INFRAFRONTIER leads creation of a service catalogue for cancer models and offers several cancer research-related services like in-depth cancer phenotyping, generation of precision cancer models, etc. In both these projects, researchers can access these services free-of-charge via the respective Open Calls.

Moving forward, INFRAFRONTIER is currently developing an even broader portfolio of disease models that includes in vitro and in silico models and thus, further strengthening its capacities for modelling human diseases. Different business models are being explored, as well as new strategies are being developed to expand the scope of quality-assured cutting-edge disease models that can be provided to the research community.

## References

[CR1] Belkaid Y, Hand TW (2014). Role of the microbiota in immunity and inflammation. Cell.

[CR2] Dabke K, Hendrick G, Devkota S (2019). The gut microbiome and metabolic syndrome. J Clin Invest.

[CR3] Ehlich H, Cater HL, Flenniken AM (2022). INFRAFRONTIER quality principles in systemic phenotyping. Mamm Genome off J Int Mamm Genome Soc.

[CR4] Gailus-Durner V, Fuchs H, Adler T (2009). Systemic first-line phenotyping. Methods Mol Biol Clifton NJ.

[CR5] Groza T, Gomez FL, Mashhadi HH (2023). The International mouse phenotyping consortium: comprehensive knockout phenotyping underpinning the study of human disease. Nucleic Acids Res.

[CR6] Hirst GL, Balmain A (2004). Forty years of cancer modelling in the mouse. Eur J Cancer Oxf Engl.

[CR7] Huijbers IJ, Del Bravo J, Bin Ali R (2015). Using the GEMM-ESC strategy to study gene function in mouse models. Nat Protoc.

[CR8] INFRAFRONTIER Consortium (2015). INFRAFRONTIER–providing mutant mouse resources as research tools for the international scientific community. Nucleic Acids Res.

[CR9] Johnson KV-A, Foster KR (2018). Why does the microbiome affect behaviour?. Nat Rev Microbiol.

[CR10] Lane-Petter W (1962). Provision of pathogen-free animals. Proc R Soc Med.

[CR11] Madeo F, Carmona-Gutierrez D, Hofer SJ, Kroemer G (2019). Caloric restriction mimetics against age-associated disease: targets, mechanisms, and therapeutic potential. Cell Metab.

[CR12] Mouse Genome Sequencing Consortium (2002). Initial sequencing and comparative analysis of the mouse genome. Nature.

[CR13] Mukherjee P, Roy S, Ghosh D, Nandi SK (2022). Role of animal models in biomedical research: a review. Lab Anim Res.

[CR14] Mulderrig L, Garaycoechea JI, Tuong ZK (2021). Aldehyde-driven transcriptional stress triggers an anorexic DNA damage response. Nature.

[CR15] Murillo-Cuesta S, Artuch R, Asensio F (2020). The value of mouse models of rare diseases: a Spanish experience. Front Genet.

[CR16] Perlman RL (2016). Mouse models of human disease: an evolutionary perspective. Evol Med Public Health.

[CR17] Raess M, De Castro AA, the INFRAFRONTIER Consortium (2016). INFRAFRONTIER: a European resource for studying the functional basis of human disease. Mamm Genome.

[CR18] Rath E, Haller D (2022). Intestinal epithelial cell metabolism at the interface of microbial dysbiosis and tissue injury. Mucosal Immunol.

[CR19] Ryder E, Doe B, Gleeson D (2014). Rapid conversion of EUCOMM/KOMP-CSD alleles in mouse embryos using a cell-permeable Cre recombinase. Transgenic Res.

[CR20] Sansone SA, McQuilton P, the FAIRsharing Community (2019). FAIRsharing as a community approach to standards, repositories and policies. Nat Biotechnol.

[CR21] Schwartz C, Moran T, Saunders SP (2019). Spontaneous atopic dermatitis in mice with a defective skin barrier is independent of ILC2 and mediated by IL-1β. Allergy.

[CR22] Sepich-Poore GD, Zitvogel L, Straussman R (2021). The microbiome and human cancer. Science.

[CR23] Shcherbakov D, Nigri M, Akbergenov R (2022). Premature aging in mice with error-prone protein synthesis. Sci Adv.

[CR24] Stein M, Elefteriou F, Busse B (2023). Why animal experiments are still indispensable in bone research: a statement by the European Calcified Tissue Society. J Bone Miner Res.

[CR25] Sung H, Ferlay J, Siegel RL (2021). Global cancer statistics 2020: GLOBOCAN estimates of incidence and mortality worldwide for 36 cancers in 185 countries. CA Cancer J Clin.

[CR26] Thomas DP, Zhang J, Nguyen N-T, Ta HT (2023). Microfluidic gut-on-a-chip: fundamentals and challenges. Biosensors.

[CR27] Winslow MM, Jacks T (2015) 9 - Genetic mouse models of cancer. In: Mendelsohn J, Gray JW, Howley PM, Israel MA, Thompson CB (eds) The molecular basis of cancer, 4th edn. Elsevier, pp 145–154.e2. 10.1016/B978-1-4557-4066-6.00009-3

[CR28] Yan X, He S, Dong D (2020). Determining how far an adult rare disease patient needs to travel for a definitive diagnosis: a cross-sectional examination of the 2018 national rare disease survey in China. Int J Environ Res Public Health.

